# Agonist dependency of the second phase access of β-arrestin 2 to the heteromeric µ-V1b receptor

**DOI:** 10.1038/s41598-021-94894-y

**Published:** 2021-08-04

**Authors:** Nuttawadee Ngamlertwong, Hiroyoshi Tsuchiya, Yuta Mochimaru, Morio Azuma, Takahiro Kuchimaru, Taka-aki Koshimizu

**Affiliations:** 1 Division of Molecular Pharmacology, Department of Pharmacology, Jichi Medical University, Tochigi, 329-0498 Japan; 2 Center for Molecular Medicine, Jichi Medical University, Tochigi, 329-0498 Japan

**Keywords:** Pharmacology, Pharmacology

## Abstract

During the development of analgesic tolerance to morphine, the V1b vasopressin receptor has been proposed to bind to β-arrestin 2 and the µ-opioid receptor to enable their interaction. However, direct evidence of such a high-order complex is lacking. Using bioluminescent resonance energy transfer between a split Nanoluciferase and the Venus fluorescent protein, the NanoBit-NanoBRET system, we found that β-arrestin 2 closely located near the heteromer µ-V1b receptor in the absence of an agonist and moved closer to the receptor carboxyl-termini upon agonist stimulation. An additive effect of the two agonists for opioid and vasopressin receptors was detected on the NanoBRET between the µ-V1b heteromer and β-arrestin 2. To increase the agonist response of NanoBRET, the ratio of the donor luminophore to the acceptor fluorophore was decreased to the detection limit of luminescence. In the first phase of access, β-arrestin 2 was likely to bind to the unstimulated V1b receptor in both its phosphorylated and unphosphorylated forms. In contrast, the second-phase access of β-arrestin 2 was agonist dependent, indicating a possible pharmacological intervention strategy. Therefore, our efficient method should be useful for evaluating chemicals that directly target the vasopressin binding site in the µ-V1b heteromer to reduce the second-phase access of β-arrestin 2 and thereby to alleviate tolerance to morphine analgesia.

## Introduction

A range of extracellular information, mediated by peptides, small molecules, chemicals, and physical stimuli, has been detected by G protein-coupled receptors (GPCRs)^1,2^. Increasing evidence suggests that in addition to a functional monomeric receptor, GPCRs work as multimeric molecular complexes, which contain more than two receptor protomers^3^. Receptor dimers or higher-order oligomers can be formed by two of the same or different types of GPCRs in a constitutive or agonist-dependent manner^4,5^. Dimerization can gain functional diversity, which is not observed when each receptor is analyzed separately^6,7^. In particular, heteromeric receptors are a focus of pharmaceutical interest as a new type of drug target^8^. However, the difficulty in analyzing receptor heteromer-mediated responses partly due to the facts that monomeric and homo-multimeric receptors can coexist with receptor heteromers in a cell, and that all receptor types can be stimulated simultaneously. While specific tools, such as dual ligands and heteromer-specific antibodies, have been developed for the analysis of heteromeric receptors, their generalization remains challenging^6,9,10^.

β-arrestin 1 and β-arrestin 2, also known as arrestin 2 and 3, respectively^11^, are non-visual arrestins. β-arrestins have a low affinity for unstimulated GPCRs. Stimulation of the receptor by an agonist and subsequent phosphorylation of the carboxyl- (C-) terminus and/or intracellular third loop of the GPCR by a G protein-coupled receptor kinase initiates the access of β-arrestin to the receptor with high affinity^12–15^. Receptor-bound β-arrestins play important roles in signal transduction, such as desensitization, internalization, and recycling of the stimulated receptors^1^. Therefore, the kinetics of the receptor-β-arrestin interaction have been extensively studied. Methods to monitor such protein–protein interactions often use fluorescence and/or bioluminescence resonance energy transfer (FRET and BRET). Compared with FRET, BRET has the advantage of low background noise, but weak donor luminescence and low spatial resolution limit its utility. A bright and small luciferase protein, Nanoluciferase (Nluc)^16^, was developed and utilized as an energy donor for BRET analysis (NanoBRET)^17^. In NanoBRET analysis, the spatial proximity between two molecules, Nluc and an energy acceptor fluorophore, can be examined. Additionally, interactions between two molecules can be analyzed by splitting Nluc into two complementary parts, a large part (LgBit) and a small part (SmBit), and by connecting them to each protein of interest to examine proximity (NanoBit)^18^. However, biological systems are frequently composed of complexes with multiple components^3^. The direct examination and complete elucidation of complexes composed of these three molecules, receptor dimers and β-arrestin, remain limited.

Acute pain can be managed by the use of opioid analgesics, such as morphine. However, repeated or continuous use of opioids may lead to tolerance, after which the opioid dose needs to be increased to achieve the same analgesic effect. Morphine tolerance is developed by changes at the molecular, cellular, neural network, and metabolic levels^19,20^. At the receptor level, C-terminal phosphorylation and β-arrestin recruitment are critical parameters underlying morphine tolerance^21^, although other mechanisms can participate in this process^22^. We previously reported that the mouse V1b receptor recruited β-arrestin 2 to the leucine-rich segment in the V1b C-terminus in the absence of an agonist^23^. This V1b-β-arrestin 2 complex was proposed to enhance intracellular signals thereby leading to analgesic tolerance to morphine in cells expressing both V1b and μ-opioid (μ) receptors^23–25^. However, questions remain regarding the V1b-β-arrestin 2 complex. First, the agonist did not change the apparent interaction between V1b and β-arrestin 2 when the BRET efficiency between V1b-Nluc and β-arrestin 2-Venus was monitored. In contrast, the other members of the vasopressin receptor family, V1a and V2 subtypes, recruited β-arrestin 2 in an agonist-dependent manner^26^. Second, the formation of a molecular complex composed of β-arrestin 2, V1b, and μ receptors has been proposed, but direct evidence has not been obtained.

Here, we report the use of a NanoBit split Nluc and NanoBRET to sensitively monitor the access of β-arrestin 2 to the homodimeric V1b receptor and the heterodimeric μ-V1b receptor. Reducing the receptor-Nluc/β-arrestin 2-Venus ratio by more than 100-fold markedly increased the BRET signal upon transfection, leading to the detection of agonist-dependent V1b activation. The improvement was also achieved in the V1a and μ receptors, indicating the broad application of our method to the analysis of β-arrestin 2-dependent signaling pathways.

## Results

### Agonist-dependent and -independent interactions between the homodimeric V1b receptor and β-arrestin 2

**Figure 1:**
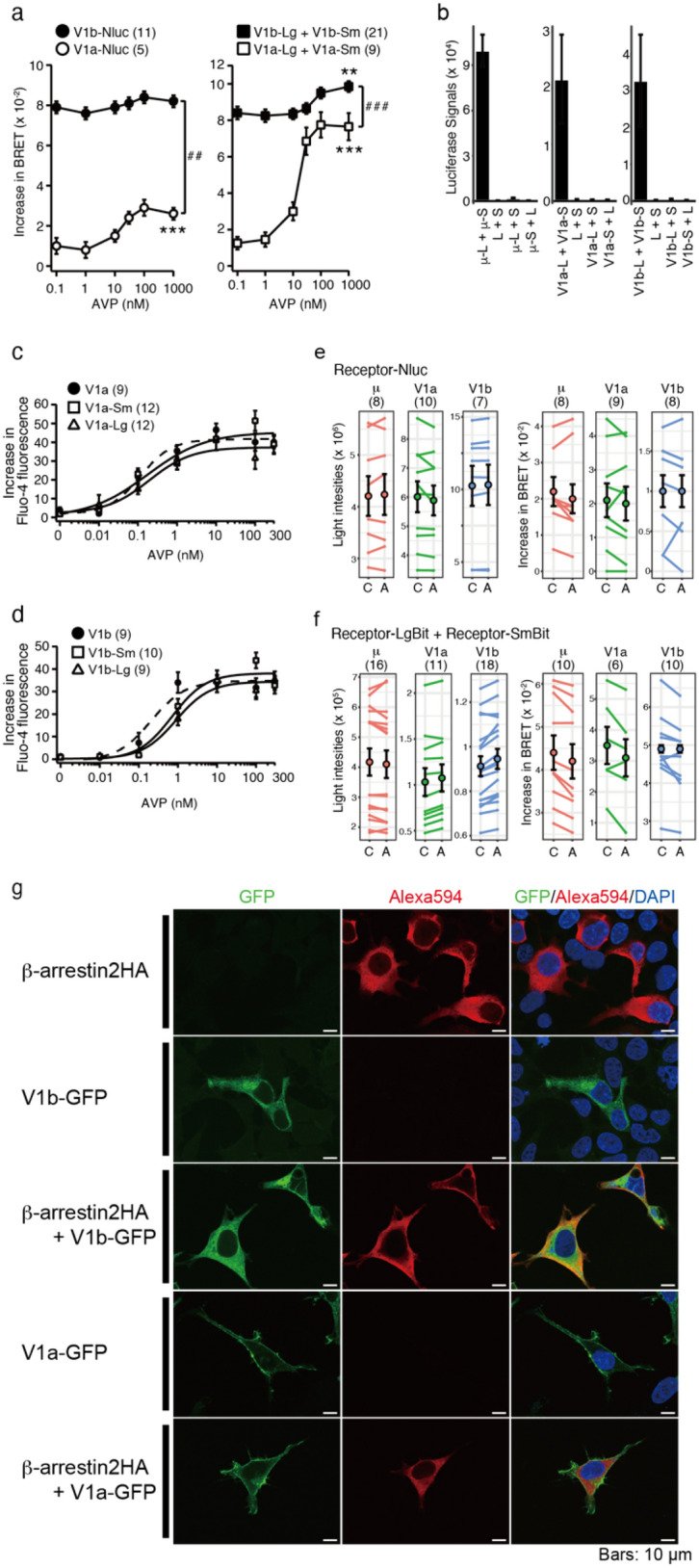
NanoBRET analysis detected the agonist-dependent access of β-arrestin 2 to V1a and V1b receptors. (**a**) A whole Nanoluciferase (left panel) or each part of a split Nanoluciferase (right panel) was fused to the C-termini of V1a and V1b receptors. The access of β-arrestin 2-Venus to the receptors was monitored by BRET measurement after AVP stimulation. ## and ### indicate that differences between V1a and V1b receptors are significant. ##, *p* < 0.01 and ###, *p* < 0.001. * and ** indicate significant increases due to AVP. *, *p* < 0.01 and **, *p* < 0.001. (**b**) Large and small NanoBit components (L or S) were fused to the C-termini of V1a, V1b, and μ receptors for expression in HEK cells. Only cells that expressed homodimeric receptors showed strong luminescence signals. n = 6, 3, and 3 for the V1a, V1b and μ receptors, respectively. (**c**,**d**) HEK cells were transfected with the indicated plasmid constructs encoding V1a (**c**) and V1b (**d**) receptors, and the AVP-induced intracellular Ca^2+^ responses were monitored. (**e**,**f**) Neither AVP or DAMGO stimulation (1 μM, A) nor buffer treatment (**c**) changed luminescence signals or the luminescence intensity ratio at 530/480 nm in cells expressing receptor-Nluc (**e**) or the receptor-fused to LgBit or SmBit (**f**). In (**e**,**f**), the cells were not transfected with plasmids encoding β-arrestin 2-Venus. (**g**) Cellular localizations of V1b-GFP and β-arrestin 2. V1b- GFP or V1a-GFP was coexpressed with β-arrestin 2-HA in HEK cells. GFP was visualized as green fluorescence, while the HA tag was detected by a rat anti-HA antibody followed by an anti-rat Alexa594 secondary antibody. Nuclei were stained with DAPI. The scale bars represent 10 mm. The data were obtained from a representative experiment that was performed in triplicate. The statistical computer program R version 4.0.3 (R Core Team, [2020], Vienna, Austria. https://www.r-project.org) was used to prepare Fig. 1e.

Concentration–response curves constructed from the NanoBRET measurements of receptor-Nluc and β-arrestin 2-Venus, revealed marked differences between V1a and V1b (Fig. 1a, left). The interaction between V1a-Nluc and β-arrestin 2-Venus was initiated by agonists and was increased in an agonist-dependent manner. In contrast, the basal NanoBRET signal in the absence of an agonist was already high in cells expressing both V1b-Nluc and β-arrestin 2-Venus, indicating that the interaction occurred without agonist stimulation (Fig. 1a, left). Furthermore, agonist-dependent increases in NanoBRET were not detected between V1b-Nluc and β-arrestin 2-Venus. However, we and others previously reported V1b receptor-initiated increases in intracellular Ca^2+^ and ERK phosphorylation levels upon arginine vasopressin (AVP) stimulation^26^, indicating that the agonist stimulation of V1b in the cell surface may involve further conformational changes in the V1b-β-arrestin 2 complex. Therefore, we explored an experimental condition in which two parts of a split Nluc (LgBit and SmBit) were fused to the C-terminus of V1b or V1a, and the interaction between the homodimer receptor and β-arrestin 2 was examined using the NanoBit-NanoBRET system. In this system, AVP significantly increased the NanoBRET signal in both V1a and V1b homodimers (Fig. 1a, right panel), whereas basal interaction without an agonist was detected only in the V1b homodimer and β-arrestin 2. The C-terminal fusion of small or large parts of split Nluc enabled the detection of homodimer receptors (Fig. 1b) and did not affect V1a or V1b receptor functions in terms of the intracellular Ca^2+^ responses (Fig. 1c,d). Moreover, agonist stimulation did not change the luminescence intensity or the BRET 530/480 nm ratio when β-arrestin 2-Venus was excluded from the transfected DNA, indicating that the agonist-dependent changes in the NanoBRET signals were not caused by changes in the luminescence signals from the receptor dimers (Fig. 1e,f). When cellular localization of V1b-GFP and β-arrestin 2HA was visualized, both signals were detected intracellularly, which was consistent with a constitutive interaction. V1b-GFP was also detected in the plasma membrane (Fig. 1g). In contrast, V1a-GFP was mainly distributed in the plasma membrane, while β-arrestin 2HA was distributed in the cytoplasm (Fig. 1g). It should be noted that although substantial amounts of V1b receptors were detected intracellularly, the cell surface V1b receptors were functional and responded to AVP, when AVP was applied into external buffer (Fig. 1a,d).

In our NanoBRET measurements obtained using Nluc and Venus, the energy transfer was low between distinct receptor families, such as between seven-transmembrane μ receptors and two-transmembrane P2X2a purinergic receptor channel subunits^27^ and between V1a and P2X2a (Supplemental Figure S1), indicating that the membrane-limited localization of receptors was not sufficient for positive NanoBRET signals. In cells expressing receptor-fused split Nluc, injection of the luminescent substrate into the cellular suspension increased the luminescence signal over time (Supplemental Figure S2a). This gradual increase was not caused by limited access of the substrate to the intracellular space, because after preparation of the membrane fraction, a receptor dimer, V1a-LgBit or V1a-SmBit, directly accessed the substrate and generated luminescence signals with gradually increasing intensity (Supplemental Figure S2b). The luminescence signal was gradually increased after the addition of the luminescence substrate in constitutively associated cAMP-dependent protein kinase type II-alpha regulatory subunit and catalytic subunit pairs (Supplemental Figure S2c). These results suggested that the substrate may have increased the activity of Nluc, which was comprised of LgBit and SmBit. In this study, we averaged the luminescence intensities obtained during a measurement period of five min.

### Individual and combined effects of a stimulating heterodimer by both agonists on NanoBRET

**Figure 2:**
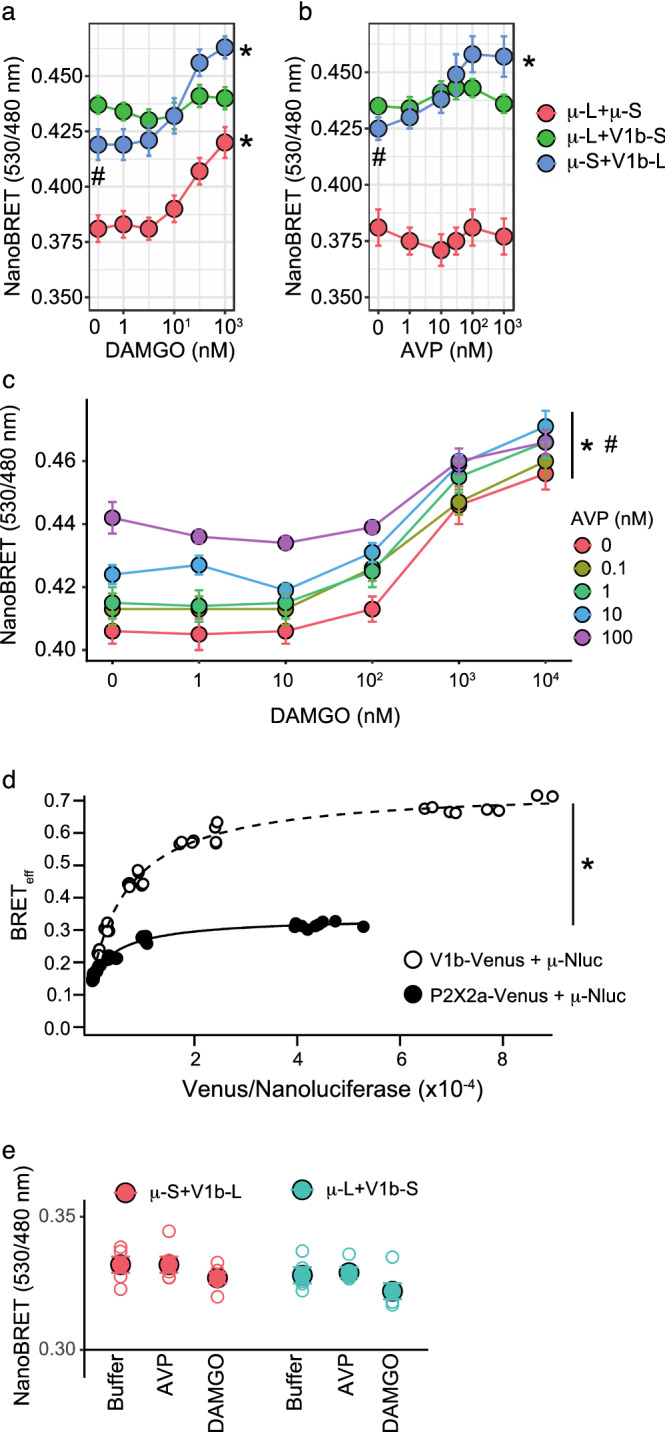
Agonists for μ and V1b receptors stimulated the access of β-arrestin 2 to μ-V1b receptors. NanoBit components, LgBit (L) and SmBit (S), were fused to the μ and V1b receptors. Plasmid DNAs (0.75 μg for each receptor construct) were coexpressed in HEK cells together with 3 μg of β-arrestin 2-Venus and the cells were stimulated with the indicated concentrations of (**a**) DAMGO (n = 8), (**b**) AVP (n = 6–7) and (**c**) DAMGO plus AVP (n = 6). In (**c**), V1b-LgBit and μ-SmBit were used. *in (**a**,**b**) indicates a significant increase in the NanoBRET from the lowest agonist concentration (*p* < 0.05). # in (a) and (**b**) indicates a significant increase in the basal NanoBRET signal intensity (*p* < 0.05). *and # in (**c**) indicate that AVP and DAMGO were significant determinants of the NanoBRET values (*p* < 0.05). (**d**) A reduction of donor luminescence increased the NanoBRET signal in μ-V1b receptors. HEK cells were cotransfected with V1b-Venus (open circle) or P2X2a-Venus (closed circle) together with μ-Nluc at different ratios (1:0.08–39). The NanoBRET values, the fluorescence at 530 nm, and the luminescence at 480 nm, were measured. For BRET_max_, Venus and Nluc were sequentially fused to the C-terminus of the μ-receptor. The μ-Venus-Nluc construct was expressed in HEK cells, and NanoBRET signals were measured without agonist stimulation. The NanoBRET efficiency (BRET_eff_) was calculated as BRET/BRET_max_^28^. Representative data from a single experiment are shown. Experiments were repeated three times and yielded similar results. *indicates that the transfected plasmids were significant determinants of BRET_eff_ values (*p* < 0.05). (**e**) Agonists did not change the luminescence of the split Nanoluciferase, which was fused to the μ-V1b receptors. μ-LgBit + V1b-SmBit or μ-SmBit + V1b-LgBit was expressed in HEK cells and the luminescence was measured in the presence of buffer, 100 nM AVP or 100 nM DAMGO. The NanoBRET ratio at 530/480 nm was calculated. The statistical computer program R version 4.0.3 (R Core Team, [2020], Vienna, Austria. https://www.r-project.org) was used to prepare Fig. 2a–c,e.

We next examined the access of β-arrestin 2 to a heteromer receptor. The V1b or μ receptors was fused to LgBit or SmBit. Since these split Nluc components have asymmetrical structures, the NanoBRET efficiency was compared by swapping the two components that were fused to the receptors. As shown in Fig. 2a,b, the combination of V1b-LgBit and μ-SmBit was more efficient at detecting NanoBRET between the heteromer receptor and β-arrestin 2 than the opposite combination of V1b-SmBit plus μ-LgBit upon agonist stimulation of either the V1b receptor or the μ receptor. The basal NanoBRET signals were consistently increased in the absence of an agonist independent of the fused Nluc component when μ was coexpressed with V1b (Fig. 2a,b). After we confirmed that V1b-LgBit/μ-SmBit combination induced agonist-dependent NanoBRET signals upon stimulation with either one of the protomers, we examined the stimulation of both receptors simultaneously. Increased concentrations of both AVP and DAMGO ([D-Ala^2^, N-MePhe^4^, Gly-ol]-enkephalin) contributed to increases in the NanoBRET signal intensity (Fig. 2c). The formation of a specific μ-V1b heteromer receptor was examined according to a previously published method^28^. Reduction of μ-Nluc donor luminescence relative to V1b-Venus fluorescence while keeping the total expression constant, resulted in an increase in NanoBRET efficiency (BRET_eff_, Fig. 2d), and the values fitted well to the hyperbolic equation. A modified μ-receptor, which was fused to Venus and Nluc sequentially at the C-terminus (μ-Venus-Nluc), was used as a positive control. The maximum BRET value attained using μ-Venus-Nluc was 2.62 ± 0.003 (n = 10), which was set as the BRET_max_^28^. Regarding receptor pairs with low levels of interaction, P2X2a-Venus and μ-Nluc were examined. The BRET_eff_ value of μ-V1b was significantly higher than that obtained from cells coexpressing P2X2a-Venus and μ-Nluc (Fig. 2d). When receptor heteromers fused to split Nluc were expressed in the absence of β-arrestin 2-Venus, the luminescence intensities were significantly higher than the background level, indicating constitutive dimer formation. The luminescence intensities of μ-LgBit + V1b-SmBit and μ-SmBit + V1b-LgBit were 171,286 ± 19,255 and 116,108 ± 2319, respectively (n = 5). However, the luminescence intensity ratio at 530/480 nm was not changed following agonist stimulation (Fig. 2e). Therefore, the NanoBRET levels in cells expressing the μ-V1b heteromer and β-arrestin 2 likely reflected changes in the interaction between the heteromer receptor and β-arrestin 2 upon agonist stimulation.

The combination of μ receptor-Nluc and the native V1b receptor or the opposite combination of V1b-Nluc plus the native μ receptor possibly resulted in the combination of the heteromeric receptor with whole Nluc. However, costimulation with AVP and DAMGO did not change the NanoBRET responses (Supplemental Figure S3) when receptor-Nluc was used under the same transfection conditions employed in the experiments shown in Fig. 2.

### Reduced receptor-Nanoluciferase expression resulted in the sensitive detection of BRET signals generated by β-arrestin 2-Venus

Initially, transient transfection was optimized to generate a sufficient luciferase signal; therefore, 1.5 μg and 0.75 μg of the β-arrestin 2-Venus and V1b-Nluc plasmids, respectively, were transfected into 10^5^ cells plated in a 35 mm dish. However, agonist stimulation of V1b-Nluc with 1–100 nM AVP did not change the NanoBRET signal under these conditions, as reported previously^23^. Therefore, we looked for more sensitive conditions for detecting probable changes in NanoBRET signals between β-arrestin 2-Venus and V1b-Nluc.

**Figure 3:**
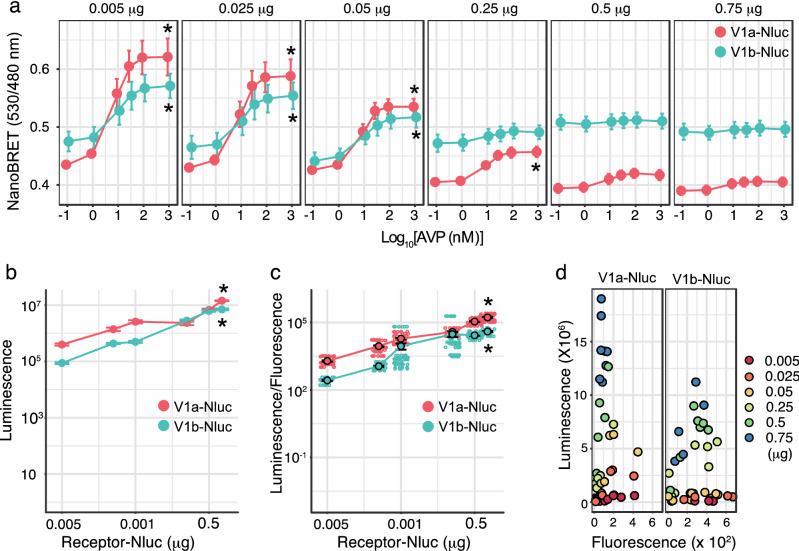
A reduction of receptor-Nluc expression increased the agonist-dependent BRET signals. (**a**) Plasmid DNAs encoding V1a-Nluc and V1b-Nluc were separately transfected at the indicated amounts together with 1.5 µg of β-arrestin 2-Venus into 10^5^ cells in a 35 mm dish. After the cells were allowed to grow for 36 to 48 h, the AVP-stimulated increase in the BRET levels was monitored. Each concentration–response curve was constructed from 5 to 9 independent experiments. *indicates a significantly increased response compared with that induced by 0.1 nM AVP (*p* < 0.05). (**b**) Increases in the transfection amount of V1a-Nluc and V1b-Nluc increased the luminescence intensities. (n = 6–9) (**c**) The ratio of Nluc luminescence/Venus fluorescence increased as the transfection amounts of the V1a- Nluc and V1b-Nluc plasmids increased. (n = 6–9) In (**b**,**c**), *indicates a significant increase compared with the response elicited by 0.005 μg of receptor-Nluc (*p* < 0.05). (**d**) Relationship between the luminescence and fluorescence intensities during the experiments shown in (**a**). (n = 6–9) The statistical computer program R version 4.0.3 (R Core Team, [2020], Vienna, Austria. https://www.r-project.org) was used to prepare Fig. 3.

A critical determinant for detecting agonist dependency in NanoBRET between V1b-Nluc and β-arrestin 2-Venus was the expression level of V1b-Nluc. Reducing the amount of V1b-Nluc plasmid transfected to 0.7% of that in the initial reaction significantly increased the agonist-dependent NanoBRET signal (Fig. 3a). Agonist-dependent access of β-arrestin 2-Venus to V1a-Nluc was detected at even higher expression levels, and the amplitude of the response increased after the expression level of V1a-Nluc was reduced (Fig. 3a). The lowest amount of the receptor-Nluc plasmid for transfection was set to produce a detectable level of specific luciferase signal. Under our expression conditions, the luminescence intensity increased as the receptor-Nluc expression level increased (Fig. 3b). When the expression levels of receptor-Nluc were examined by western blot analysis, specific signals of HA-tagged μ-Nluc were detected in cells transfected with the second largest amount of the plasmid (Supplemental Figure Fig. S4, lane 5). The HA-tagged μ-Nluc exhibited the expected molecular weight of 72.3 kDa as determined by western blot analysis. The calculated ratio of the Nluc luminescence intensity to the Venus fluorescence intensity increased as the amounts of plasmid transfected increased (Fig. 3c). These results indicated that our expression conditions did not reach saturation and that the expression levels were controlled by the amount of the receptor-Nluc plasmid transfected. We used a fixed amount of the β-arrestin 2-Venus plasmid in the experiment shown in Fig. 3a. However, the fluorescence intensities of β-arrestin 2-Venus decreased as the receptor-Nluc expression increased (Fig. 3d).

**Figure 4:**
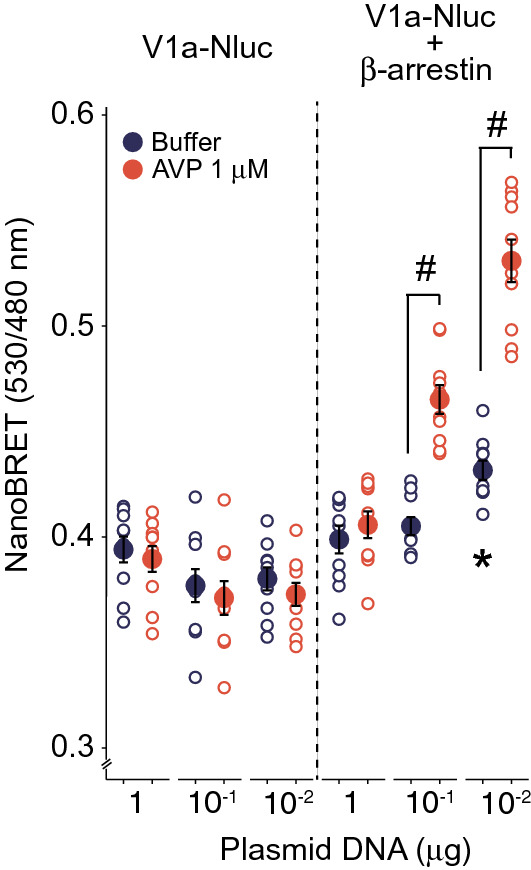
β-arrestin 2 was necessary for increasing the NanoBRET signal in cells expressing V1a-Nluc. Cells growing on a 35 mm dish were transfected with different amounts of the V1a-Nluc plasmid with or without the β-arrestin 2-Venus plasmid (1.5 µg). The NanoBRET signals were measured after treatment with 1 µM AVP or buffer. (n = 10) *indicates that the basal BRET level was increased by V1a-Nluc (0.01 µg) plus β-arrestin 2-Venus (1.5 µg; *p* < 0.05). # indicates that the NanoBRET was increased following AVP stimulation (*p* < 0.05). The statistical computer program R version 4.0.3 (R Core Team, [2020], Vienna, Austria. https://www.r-project.org) was used to prepare Fig. 4.

In V1a-Nluc-expressing cells, the NanoBRET values by 0.1 nM AVP gradually increased as the expression of V1a-Nluc decreased (Fig. 3a). β-Arrestin 2-Venus was necessary for the increased NanoBRET intensity in these cells (Fig. 4). Reduced expression of V1a-Nluc and increased expression of β-arrestin 2 increased the NanoBRET levels (Fig. 4). The BRET ratio at 530/480 nm did not change over a wide range of expression levels when the plasmid for β-arrestin 2-Venus was not included in transient expression (Fig. 4, left panel).

### Reduced expression of µ-V1b receptor enabled the sensitive detection of an agonist-induced increase in NanoBRET between the receptor and β-arrestin 2

**Figure 5:**
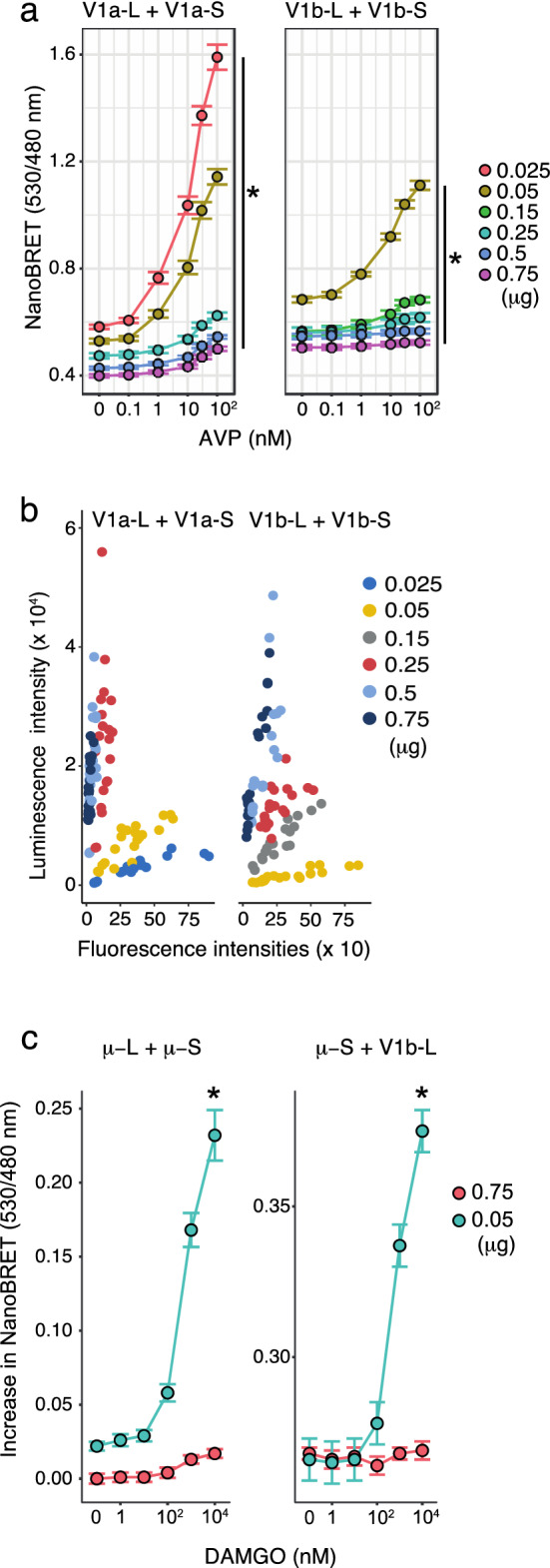
Reduced expression levels of the heteromeric receptors increased the intensity of the NanoBRET signals between heteromeric receptors and β-arrestin 2. (**a**) NanoBRET measurements were performed to monitor a complex formation among receptor homodimer and β-arrestin 2. Cells were transfected with the indicated amounts of receptor-LgBit and receptor-SmBit plasmids and a fixed amount (1.5 μg) of β-arrestin 2-Venus. Reduced plasmid amounts of the receptor-LgBit and receptor-SmBit significantly increased the NanoBRET signals. (n = 10) *indicates that the plasmid amount of receptor constructs was a significant determinant in the BRET responses (*p* < 0.001). (**b**) Relationship between the luminescence from receptor-homomer-NanoBit and the fluorescence from β-arrestin 2-Venus. The plasmid amounts of the receptor constructs in a 35 mm dish are indicated. (n = 10) (**c**) The homodimeric μ-receptor and heterodimeric μ-V1b receptor recruited β-arrestin 2-Venus with a large NanoBRET signal when the expression levels of the receptors were reduced. The indicated amounts of the receptor plasmids were cotransfected with 1.5 μg of β-arrestin 2-Venus into cells growing in a 35 mm dish. (n = 10) *indicates that reduced amounts of the receptor plasmids significantly increased the BRET responses (*p* < 0.001). The statistical computer program R version 4.0.3 (R Core Team, [2020], Vienna, Austria. https://www.r-project.org) was used to prepare Fig. 5.

In addition to receptors fused to whole Nluc, the expression levels of homomeric and heteromeric receptors fused to split Nluc were also key to successfully monitor agonist-induced access of β-arrestin 2. Reduced expression of the homomeric V1a or V1b receptor increased the amplitude of AVP-dependent NanoBRET (Fig. 5a). The luminescence intensities and receptor expression levels of V1a were higher than those of V1b when equal amounts of the plasmids were transfected. At the lowest amount of transfection plasmid (0.025 μg/35 mm dish), the V1b homomeric dimer did not emit a specific luminescence signal, and these conditions were excluded from the analysis. When we examined NanoBRET upon the transfection of various amounts of the V1a or V1b plasmids, a fixed amount of the β-arrestin 2-Venus plasmid (1.5 μg/35 mm dish) was used. However, Venus fluorescence intensity tended to be higher when the receptor dimer was cotransfected at a low level (Fig. 5b). These results indicated that the relative amounts of the receptor and β-arrestin 2 expressed might have substantial influence on the efficiency of NanoBRET. The importance of the expression levels of the receptor fused to split Nluc was not limited to V1 type vasopressin receptors. The expression levels of homomeric μ-receptor and heteromeric μ-V1b receptor critically regulated the NanoBRET responses (Fig. 5c).

### The β-arrestin 2 expression level also determines the amplitude of the NanoBRET signal

**Figure 6:**
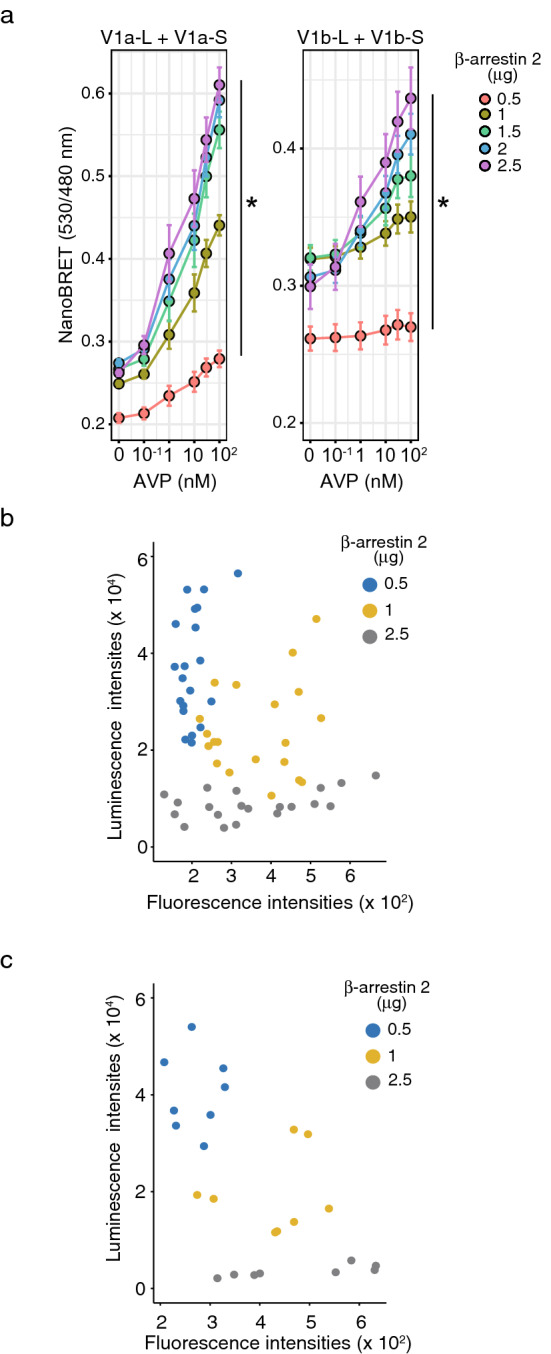
Increasing the expression level of β-arrestin 2-Venus increased the NanoBRET signals in cells expressing the homodimeric V1a or V1b receptor. (**a**) Indicated amounts of the β-arrestin 2-Venus plasmids were cotransfected with V1a-LgBit plus V1a-SmBit or V1b-LgBit plus V1b-SmBit. NanoBRET signals were examined after AVP stimulation at the indicated concentrations. (n = 8 and 6 for the V1a and V1b dimers, respectively) *indicates that the β-arrestin 2-Venus plasmid amounts were significant determinants for the BRET responses (*p* < 0.05). (**b,c**) A fixed amount (0.05 μg) of V1a-LgBit plus V1a-SmBit (**b**) or V1b-LgBit plus V1b-SmBit (**c**) was cotransfected with the indicated amount of β-arrestin 2-Venus. The luminescence and fluorescence intensity ratios were examined from 36 to 48 h after transfection. (n = 20 and 8 for the V1a and V1b dimers, respectively) The statistical computer program R version 4.0.3 (R Core Team, [2020], Vienna, Austria. https://www.r-project.org) was used to prepare Fig. 6.

We next examined the effect of increasing the expression level of β-arrestin 2-Venus while fixing the transfection amounts of the receptor-LgBit and receptor-SmBit plasmids. Increasing the expression of β-arrestin 2 increased the maximum BRET responses in cells expressing homomeric V1a or V1b receptors (Fig. 6a). The basal NanoBRET levels in cells expressing the homomeric V1b receptor were higher than those in cells expressing homomeric V1a receptor in the absence of AVP. This result indicated that the V1b homodimers were more likely to bind with β-arrestin 2 under basal conditions. The luminescence intensities of receptor dimers tended to be low if the expression of β-arrestin 2-Venus was high (Fig. 6b,c), resulting in a high amplitude of the agonist-induced NanoBRET signal.

## Discussion

In this study, we demonstrated the formation of a three-molecule complex, composed of the μ, V1b receptors and β-arrestin 2, using NanoBit and NanoBRET technologies. Two types of agonists for μ and V1b receptors additively increased this interaction. Furthermore, for two types of donor luminophores, whole and split Nluc, the agonist-induced response in NanoBRET was increased by decreasing the protein expression ratio of receptor-donor/βarrestin 2-acceptor. Because activation of the μ-V1b receptor heteromer and β-arrestin 2 was suggested to drive adenylate cyclase sensitization and morphine tolerance^23^, our new method for analyzing the three-molecule NanoBRET could be useful for screening therapeutic candidates capable of alleviating morphine tolerance.

The increase in the agonist-mediated NanoBRET signals upon decreasing the donor luminophore is a novel finding of our study. This increase in the agonist effect was likely due to specific interactions between the donor luminophore and the acceptor fluorophore^28^. Because luciferases and fluorescent proteins were fused to the C- or N-terminal ends of the proteins examined, we were detecting only interactions between these parts of the molecules. GPCR dimers are formed through interactions between transmembrane domains or between C-termini in a case of class C GPCRs. Our new finding is that though the monitoring C-terminal interactions between V1b and μ receptors, agonist effect of recruiting β-arrestin was detected from simultaneous stimulation of both sides of the receptor agonists. It is not known at present whether μ and V1b receptors form a receptor dimer though the transmembrane domains. To achieve a better NanoBRET response, the amount of receptor-Nluc plasmid transfected was decreased to 0.7% of the optimum expression. The brightness of Nluc made it possible to detect even a low expression under this condition. The intensity of the luminescence increased in relation to a wide range of expression levels in this study. When we performed radioligand binding studies on cells expressing the lowest levels of V1b-Nluc, specific binding was not detectable, probably due to the low expression levels (data not shown). The high β-arrestin 2 level relative to that of V1b in our experiment might not correctly simulate the conditions in native tissues. However, our sensitive method for measuring NanoBRET signals is clearly advantageous for screening partial agonists and antagonists that target the AVP binding site in the μ-V1b receptor.

The following points were evaluated when NanoBit and NanoBRET technologies were applied to the analysis of three-molecule interactions. First, the intrinsic affinity between LgBit and SmBit was reported to be 190 μM, and its effect was evaluated^18^. To prevent the detection of artificial interactions, we first evaluated interactions between two constructs fused to Nluc and Venus in a preliminary experiment and in a previous study^23^ because intrinsic affinity was not reported for this pair of molecular tags. Second, during agonist stimulation and receptor-receptor interactions, the wavelength of luminescent light and the BRET signal at 530/480 nm may change even without β-arrestin 2-Venus. However, this was not the case in our experiments. The interactions between the receptor C-termini were stable in homomeric V1a, V1b, and μ receptors and heteromeric μ-V1b receptors. Therefore, the agonist-induced changes in the cellular NanoBRET levels indicated interactions between the receptor and β-arrestin 2. Third, ligand-dependent β-arrestin recruitment to GPCRs could be followed by the bystander BRET method^29^, in which membrane-anchored GFP is used as a luminescence acceptor. We evaluated this possibility by expressing a two-transmembrane P2X ion channel fused to Venus as a bystander^30^. However, the NanoBRET values between V1a-Nluc or μ-Nluc and P2X2a-Venus were very low levels compared to those between μ-Nluc and V1b-Venus. In addition, although cellular autofluorescence might have an influence on basal NanoBRET, the effect of such autofluorescence should be equal in all experimental conditions.

Both types of the heteromeric μ-V1b receptor agonists enhanced the access to β-arrestin 2, indicating that antagonists acting on the binding sites of opioid and vasopressin in the μ-V1b receptor could reduce further access and signal of β-arrestin 2. Therefore, our study highlighted the usefulness of antagonists specific to the heteromeric μ-V1b receptor, if available. Such antagonists can spare the μ receptor monomers and homomultimers, which function as receptors for the morphine analgesia^31^. Another strategy for reducing the development of tolerance is an antagonist or partial agonist that can block the binding of AVP to the μ-V1b receptor. A previous study highlighted the importance of the signaling pathway downstream of the μ-V1b receptor^23^. Genetic deletion of the whole V1b or specific deletion of the V1b C-terminus, knockdown of β-arrestin 2, and inhibition of ERK phosphorylation reduced morphine tolerance in both in vivo and in vitro models. Moreover, administration of a V1b antagonist to the rostral ventromedial medulla of mice effectively increased morphine analgesia and delayed the development of morphine tolerance^23^. Therefore, the V1b receptor in the μ-V1b dimer is an effective drug target.

We found that β-arrestin 2 binds to the μ-V1b dimer prior to agonist stimulation and that β-arrestin 2 access was further promoted by agonist stimulation. Results from structural and biochemical studies suggests that the access of β-arrestin to GPCRs is achieved via multistep processes^32,33^. Previous reports also indicated that β-arrestin 2 binds to GPCRs without receptor activation or phosphorylation^34–38^. As we observed that some of the V1b receptors were phosphorylated without agonists^23^, β-arrestin 2 bound to both phosphorylated and unphosphorylated V1b receptors without agonists, leading to high basal NanoBRET values. In contrast, cells expressing the V1a and μ receptors showed relatively low basal NanoBRET values, which might have been due to β-arrestin 2 binding to unphosphorylated receptors. The vasopressin stimulation of V1b receptors likely increased the phosphorylated and activated forms of V1b receptors and β-arrestin access. Constitutive activity represents one possible explanation for the phosphorylation of V1b receptors without agonists. Constitutively active mutant V2 receptors have been shown to be phosphorylated and internalized without agonist^39^. We demonstrated the intracellular localization of V1b receptors without agonist stimulation in this study and in previous studies^40^. However, the hypothesis that the V1b receptor has constitutive activity requires experimental proof. Agonist-independent GPCR phosphorylation represents another possibility by which V1b phosphorylation is achieved without stimulation, as this phenomenon can occur in transfected HEK cells expressing GRK4/5/6 subfamily members^41^. However, Li et al. showed that the endogenous expression levels of GRK 5 and 6 in HEK293 cells were not sufficiently high to phosphorylate inactive receptors. When inactive rhodopsin in retinal rod disks was phosphorylated by GRK1, an initial interaction between activated rhodopsin and GRK1 was required. Dense expression of rhodopsin and diffusion of GRK1 resulted in inactive rhodopsin phosphorylation around the activated rhodopsin^42^. In the case of unstimulated V1b, we previously reported that deletion of the receptor C-terminus revealed an overlap between the amino acids necessary for phosphorylation and those necessary for interaction with β-arrestin 2^23^, indicating the importance of phosphorylation of the receptor C-terminus for β-arrestin 2 recruitment. The chemokine receptor CC1R and decoy receptor D5 were reported to constitutively bind with β-arrestin without agonists^37,43^.

Structural and functional characterization of GPCRs and arrestins suggests that one receptor possibly interacts with one arrestin molecule in rhodopsin, M2 muscarinic, NT1 neurotensin, and mutant β1 adrenergic receptors^14,15,33,44–46^. The central “finger” loop of the arrestin molecule inserts into the central cavity of the receptor^33^. The bulkiness of β-arrestin relative to the shallow cytoplasmic face of the GPCR monomer indicates that only one protomer of the receptor dimer can interact with β-arrestin. In this configuration arrestin interaction with two GPCR protomers in a receptor dimer at the same time seems impossible. From our previous results, deletion of V1b C-terminus resulted in a loss of basal BRET between μ-Nluc and β-arresin-Venus^23^. Therefore, in the case of μ-V1b- m heteromer, we assume that β-arrestin binds to the V1b C-terminus constitutively, but not to the μ receptor. We directly demonstrated, using asymmetrical components of split Nluc, that LgBit should be connected to the V1b receptor C-terminus to have a large NanoBRET signal, which was generated by interaction with β-arrestin 2-Venus. Furthermore, the effect of the two types of agonists on the V1b-LgBit-μ-SmBit dimer indicated that both vasopressin and opioid agonists enabled the movement of β-arrestin 2 closer to the V1b-LgBit. Our previous data on NanoBRET signals between μ-Nluc and β-arrestin 2-Venus in the presence of a native V1b receptor also suggested an indispensable role of the V1b C-terminus; when the V1b receptor C-terminus was deleted to remove a stretch of a leucine-rich segment, the NanoBRET between μ and β-arrestin 2 was significantly suppressed^23^.

In summary, the NanoBit and NanoBRET analysis described herein revealed a three-molecule complex made by agonist-induced access of β-arrestin 2 to the μ-V1b receptor. A similar method was recently reported to demonstrate the interaction between the Gi-β-arrestin 2 complex and the V2 receptor^47^. Agonist-dependent NanoBRET responses between homomeric or heteromeric receptors were increased by reducing the luminescence donor/acceptor ratio. Such sensitive measurements should be useful in the search for antagonists targeting V1b in the μ-V1b receptor heteromer. Even under the condition of constitutive β-arrestin 2 access to the V1b receptor, competitive or noncompetitive antagonists of V1b can be selected by monitoring the enhanced NanoBRET response. Moreover, partial agonists of the μ-V1b-β-arrestin 2 pathway can be searched under the high maximum response of the full agonist AVP. Therefore, this study provides versatile methods for screening chemicals that can reduce morphine tolerance.

## Methods

### Materials

The Venus fluorescent protein gene was provided by Dr. A. Miyawaki (RIKEN, Japan)^48^. Morphine hydrochloride was purchased from Takeda Pharmaceutical. [Arg^8^]-Vasopressin was purchased from Peptide Institute, Inc. (Osaka, Japan). DAMGO was purchased from Sigma-Aldrich (St. Louis, MO, USA). Fluo-4 acetoxymethyl ester was purchased from Thermo Fisher Scientific (Tokyo, Japan). The FuGENE HD, NanoGlo Luciferase Assay System, pNL1.1[Nluc] vector, and NanoBit PPI Starter System were purchased from Promega (Tokyo, Japan). The Plasmid Midi Kit was obtained from QIAGEN. *Escherichia coli* DH5α competent cells were obtained from Takara Bio (Kusatsu, Japan). Restriction enzymes were obtained from Takara Bio and New England Biolabs Japan, Inc. (Tokyo, Japan). The anti-HA antibody was purchased from Sigma-Aldrich. All other chemicals were of reagent grade (Wako Pure Chemical Industries, Osaka, Japan).

### Plasmid construction

The coding sequence of LgBit was amplified from the pBiT1.1-C[TK/LgBiT] vector by PCR and inserted into the KpnI/HindIII site of the pcDNA3.1(-) vector to generate pcDNA-LgBit. Oligonucleotides containing the SmBit sequence 5′-CGTGACCGGCTACCGGCTGTTCGAGGAGAT-3′ and 5′-AGCTTATCGATTTACAGAATCTCCTCGAAC-3′ were synthesized for linker ligation. Their 5′ ends were phosphorylated and the linker was inserted into the KpnI/HindIII site of pcDNA3.1(-), generating pcDNA-SmBit. The LgBit and SmBit sequences in the pcDNA vector were fused to the C-termini of the mouse μ, V1a and V1b receptors, by inserting the receptor coding sequences without a stop codon into the EcoRI/KpnI, EcoRI/BamHI, and EcoRI/BamHI sites, respectively^23^. When V1b-LgBit was prepared, the stop codon was removed, and an additional glycine sequence, encoded by GGT, was inserted. All nucleotide sequences were confirmed using PCR. The method for constructing the β-arrestin 2-Venus expression plasmid was previously described^23^.

### Cell culture and transfection

Human embryonic kidney (HEK) cells were obtained from the RIKEN BioResource Center (Ibaraki, Japan) and grown in Dulbecco’s modified Eagle’s medium (DMEM) supplemented with 10% heat-inactivated fetal bovine serum, 100 units/mL penicillin, and 100 μg/mL streptomycin. The cells were cultured at 37 °C in 5% CO_2_ in an air-ventilated humidified incubator and passaged using 0.05% trypsin and 0.53 mM EDTA. For BRET measurements, 1 × 10^5^ cells were seeded in a 35 mm tissue culture dish. Two days after seeding the cells, the medium was supplemented with a mixture of 0.2 mL of serum-free DMEM, 6 μL of FuGene HD transfection reagent (Promega, Tokyo, Japan), and 0.75 μg of the plasmid for receptor-LgBit and receptor-SmBit and 1.5 μg of β-arrestin 2-Venus. BRET measurements were performed at 36–48 h after transfection.

### BRET measurements

Energy transfer from Nluc to Venus was measured as previously described^23,30^. Approximately 36–48 h after the transfection, the cells were washed once with the assay buffer, containing 137 mM NaCl, 5 mM KCl, 1.2 mM CaCl_2_, 1 mM MgCl_2_, 10 mM HEPES (pH 7.4), and 10 mM glucose, suspended in 1 mL of the same buffer, and aliquoted into a 96-well plate at 90 µL/well. Ten microliters of agonist was added to the wells, and the plate was gently mixed and incubated for 5 min at ambient temperature. After incubation, 90 µL of a luminescence substrate was added to achieve a final dilution factor of 100. Luminescence intensities were measured at 480 and 530 nm using a fluorescence/luminescence microplate reader (SpectraMax M3; Molecular Devices, Sunnyvale, CA, USA) after the addition of the luciferase substrate (Promega). The BRET signal was calculated as follows: (light intensity at 530 nm)/(light intensity at 480 nm).

### Measurement of intracellular calcium responses

Single-cell intracellular calcium ion measurements were performed as previously described, with slight modifications^27^. Briefly, cells on a 35 mm glass-bottom plate were incubated with 3 μM Fluo-4 AM in the assay buffer at 37 °C for 40 min. The cells were washed once with the assay buffer. The glass-bottom dish containing cells in 0.5 mL of assay buffer was mounted on the stage of a fluorescence microscope (Nikon ECLIPSE TI-U, Tokyo). The cells were stimulated with vasopressin at a final volume of 1 mL. Calcium responses were examined under a 40 × objective during exposure to blue light, and the intensities of light emission at 480 and 520 nm were measured every 300–500 ms. The responses were analyzed using the computer program ImageJ (version 1.5.0i, National Institute of Health, USA).

### Immunocytochemistry

HEK cells on 35 mm glass-bottom dishes were transfected with cDNAs encoding V1a-GFP or V1b-GFP and β-arrestin 2HA, and cultured for 48 h. Immunological detection of β-arrestin 2HA and fluorescence detection using confocal microscopy (Olympus 1000 FV, Japan) were performed as previously described^40^.

### Statistics

All values in the text are reported as the mean ± S.E.M. Concentration–response curves were fitted to Hill’s four-parameter logistic equation using the nonlinear curve-fitting computer program Igor Pro 8 (WaveMetrics, Lake Oswego, OR, USA). Significant differences were determined by Student’s t-test or an ANOVA followed by a multiple comparison test with Holm’s adjustment. Statistical analysis was performed using the statistical computer program R version 4.0.3 (R Core Team, [2020], Vienna, Austria. https://www.r-project.org).

## Supplementary Information

Supplementary Information.

## Data Availability

The datasets generated during and/or analyzed during this study are available from the corresponding author on reasonable request. All unique/stable reagents generated in this study are available from the Lead Contact upon the completion of a Materials Transfer Agreement.

## References

[1] Gurevich VV, Gurevich EV (2019). GPCR signaling regulation: The role of GRKs and arrestins. Front Pharmacol..

[2] Wingler LM, Lefkowitz RJ (2020). Conformational basis of G protein-coupled receptor signaling versatility. Trends Cell Biol..

[3] Ferre S (2015). The GPCR heterotetramer: Challenging classical pharmacology. Trends Pharmacol. Sci..

[4] Gomes I, Ayoub MA, Fujita W, Jaeger WC, Pfleger KD, Devi LA (2016). G Protein-coupled receptor heteromers. Annu. Rev. Pharmacol. Toxicol..

[5] Moller J, Isbilir A, Sungkaworn T, Osberg B, Karathanasis C, Sunkara V, Grushevskyi EO, Bock A, Annibale P, Heilemann M, Schutte C, Lohse MJ (2020). Single-molecule analysis reveals agonist-specific dimer formation of micro-opioid receptors. Nat. Chem. Biol..

[6] Toneatti R, Shin JM, Shah UH, Mayer CR, Saunders JM, Fribourg M, Arsenovic PT, Janssen WG, Sealfon SC, Lopez-Gimenez JF, Benson DL, Conway DE, Gonzalez-Maeso J (2020). Interclass GPCR heteromerization affects localization and trafficking. Sci. Signal.

[7] Wang W, Qiao Y, Li Z (2018). New insights into modes of GPCR activation. Trends Pharmacol. Sci..

[8] Milligan G, Ward RJ, Marsango S (2019). GPCR homo-oligomerization. Curr. Opin. Cell Biol..

[9] Yekkirala AS, Lunzer MM, McCurdy CR, Powers MD, Kalyuzhny AE, Roerig SC, Portoghese PS (2011). N-naphthoyl-β-naltrexamine (NNTA), a highly selective and potent activator of µ/κ-opioid heteromers. Proc. Natl. Acad. Sci. U S A.

[10] Gupta A, Mulder J, Gomes I, Rozenfeld R, Bushlin I, Ong E, Lim M, Maillet E, Junek M, Cahill CM, Harkany T, Devi LA (2010). Increased abundance of opioid receptor heteromers after chronic morphine administration. Sci. Signal..

[11] Lohse MJ, Hoffmann C (2014). Arrestin interactions with G protein-coupled receptors. Handb. Exp. Pharmacol..

[12] Kim YJ, Hofmann KP, Ernst OP, Scheerer P, Choe HW, Sommer ME (2013). Crystal structure of pre-activated arrestin p44. Nature.

[13] Shukla AK, Manglik A, Kruse AC, Xiao K, Reis RI, Tseng WC, Staus DP, Hilger D, Uysal S, Huang LY, Paduch M, Tripathi-Shukla P, Koide A, Koide S, Weis WI, Kossiakoff AA, Kobilka BK, Lefkowitz RJ (2013). Structure of active β-arrestin-1 bound to a G-protein-coupled receptor phosphopeptide. Nature.

[14] Huang W, Masureel M, Qu Q, Janetzko J, Inoue A, Kato HE, Robertson MJ, Nguyen KC, Glenn JS, Skiniotis G, Kobilka BK (2020). Structure of the neurotensin receptor 1 in complex with β-arrestin 1. Nature.

[15] Staus DP, Hu H, Robertson MJ, Kleinhenz ALW, Wingler LM, Capel WD, Latorraca NR, Lefkowitz RJ, Skiniotis G (2020). Structure of the M2 muscarinic receptor-beta-arrestin complex in a lipid nanodisc. Nature.

[16] Hall MP, Unch J, Binkowski BF, Valley MP, Butler BL, Wood MG, Otto P, Zimmerman K, Vidugiris G, Machleidt T, Robers MB, Benink HA, Eggers CT, Slater MR, Meisenheimer PL, Klaubert DH, Fan F, Encell LP, Wood KV (2012). Engineered luciferase reporter from a deep sea shrimp utilizing a novel imidazopyrazinone substrate. ACS Chem. Biol..

[17] Machleidt T, Woodroofe CC, Schwinn MK, Mendez J, Robers MB, Zimmerman K, Otto P, Daniels DL, Kirkland TA, Wood KV (2015). NanoBRET–A novel BRET platform for the analysis of protein-protein interactions. ACS Chem. Biol..

[18] Dixon AS, Schwinn MK, Hall MP, Zimmerman K, Otto P, Lubben TH, Butler BL, Binkowski BF, Machleidt T, Kirkland TA, Wood MG, Eggers CT, Encell LP, Wood KV (2016). NanoLuc complementation reporter optimized for accurate measurement of protein interactions in cells. ACS Chem. Biol..

[19] Cahill CM, Walwyn W, Taylor AM, Pradhan AA, Evans CJ (2016). Allostatic mechanisms of opioid tolerance beyond desensitization and downregulation. Trends Pharmacol. Sci..

[20] Williams JT, Ingram SL, Henderson G, Chavkin C, Schulz S, Koch T, Evans CJ, Christie MJ (2013). Regulation of µ-opioid receptors: Desensitization, phosphorylation, internalization, and tolerance. Pharmacol. Rev..

[21] Bohn LM, Lefkowitz RJ, Gainetdinov RR, Peppel K, Caron MG, Lin FT (1999). Enhanced morphine analgesia in mice lacking β-arrestin 2. Science.

[22] Kliewer A, Schmiedel F, Sianati S, Bailey A, Bateman JT, Levitt ES, Williams JT, Christie MJ, Schulz S (2019). Phosphorylation-deficient G-protein-biased µ-opioid receptors improve analgesia and diminish tolerance but worsen opioid side effects. Nat. Commun..

[23] Koshimizu TA, Honda K, Nagaoka-Uozumi S, Ichimura A, Kimura I, Nakaya M, Sakai N, Shibata K, Ushijima K, Fujimura A, Hirasawa A, Kurose H, Tsujimoto G, Tanoue A, Takano Y (2018). Complex formation between the vasopressin 1b receptor, β-arrestin-2, and the µ-opioid receptor underlies morphine tolerance. Nat. Neurosci..

[24] Raehal KM, Schmid CL, Groer CE, Bohn LM (2011). Functional selectivity at the µ-opioid receptor: Implications for understanding opioid analgesia and tolerance. Pharmacol. Rev..

[25] Williams JT, Christie MJ, Manzoni O (2001). Cellular and synaptic adaptations mediating opioid dependence. Physiol. Rev..

[26] Koshimizu TA, Nakamura K, Egashira N, Hiroyama M, Nonoguchi H, Tanoue A (2012). Vasopressin V1a and V1b receptors: From molecules to physiological systems. Physiol. Rev..

[27] Koshimizu T, Koshimizu M, Stojilkovic SS (1999). Contributions of the C-terminal domain to the control of P2X receptor desensitization. J. Biol. Chem..

[28] James JR, Oliveira MI, Carmo AM, Iaboni A, Davis SJ (2006). A rigorous experimental framework for detecting protein oligomerization using bioluminescence resonance energy transfer. Nat. Methods.

[29] Namkung Y, Le Gouill C, Lukashova V, Kobayashi H, Hogue M, Khoury E, Song M, Bouvier M, Laporte SA (2016). Monitoring G protein-coupled receptor and β-arrestin trafficking in live cells using enhanced bystander BRET. Nat. Commun..

[30] Koshimizu TA, Ueno S, Tanoue A, Yanagihara N, Stojilkovic SS, Tsujimoto G (2002). Heteromultimerization modulates P2X receptor functions through participating extracellular and C-terminal subdomains. J. Biol. Chem..

[31] Matthes HW, Maldonado R, Simonin F, Valverde O, Slowe S, Kitchen I, Befort K, Dierich A, Le Meur M, Dolle P, Tzavara E, Hanoune J, Roques BP, Kieffer BL (1996). Loss of morphine-induced analgesia, reward effect and withdrawal symptoms in mice lacking the µ-opioid-receptor gene. Nature.

[32] Gurevich VV, Gurevich EV (2004). The molecular acrobatics of arrestin activation. Trends Pharmacol. Sci..

[33] Shukla AK, Westfield GH, Xiao K, Reis RI, Huang LY, Tripathi-Shukla P, Qian J, Li S, Blanc A, Oleskie AN, Dosey AM, Su M, Liang CR, Gu LL, Shan JM, Chen X, Hanna R, Choi M, Yao XJ, Klink BU, Kahsai AW, Sidhu SS, Koide S, Penczek PA, Kossiakoff AA, Woods VL, Kobilka BK, Skiniotis G, Lefkowitz RJ (2014). Visualization of arrestin recruitment by a G-protein-coupled receptor. Nature.

[34] Gurevich VV, Dion SB, Onorato JJ, Ptasienski J, Kim CM, Sterne-Marr R, Hosey MM, Benovic JL (1995). Arrestin interactions with G protein-coupled receptors: Direct binding studies of wild type and mutant arrestins with rhodopsin, β2-adrenergic, and m2 muscarinic cholinergic receptors. J. Biol. Chem..

[35] Sohlemann P, Hekman M, Puzicha M, Buchen C, Lohse MJ (1995). Binding of purified recombinant β-arrestin to guanine-nucleotide-binding-protein-coupled receptors. Eur. J. Biochem..

[36] Mukherjee S, Gurevich VV, Preninger A, Hamm HE, Bader MF, Fazleabas AT, Birnbaumer L, Hunzicker-Dunn M (2002). Aspartic acid 564 in the third cytoplasmic loop of the luteinizing hormone/choriogonadotropin receptor is crucial for phosphorylation-independent interaction with arrestin2. J. Biol. Chem..

[37] Galliera E, Jala VR, Trent JO, Bonecchi R, Signorelli P, Lefkowitz RJ, Mantovani A, Locati M, Haribabu B (2004). β-Arrestin-dependent constitutive internalization of the human chemokine decoy receptor D6. J. Biol. Chem..

[38] Jala VR, Shao WH, Haribabu B (2005). Phosphorylation-independent β-arrestin translocation and internalization of leukotriene B4 receptors. J. Biol. Chem..

[39] Kocan M, See HB, Sampaio NG, Eidne KA, Feldman BJ, Pfleger KD (2009). Agonist-independent interactions between β-arrestins and mutant vasopressin type II receptors associated with nephrogenic syndrome of inappropriate antidiuresis. Mol. Endocrinol..

[40] Kashiwazaki A, Fujiwara Y, Tsuchiya H, Sakai N, Shibata K, Koshimizu TA (2015). Subcellular localization and internalization of the vasopressin V1b receptor. Eur. J. Pharmacol..

[41] Li L, Homan KT, Vishnivetskiy SA, Manglik A, Tesmer JJ, Gurevich VV, Gurevich EV (2015). G protein-coupled receptor kinases of the GRK4 protein subfamily phosphorylate inactive G protein-coupled receptors (GPCRs). J. Biol. Chem..

[42] Binder BM, O'Connor TM, Bownds MD, Arshavsky VY (1996). Phosphorylation of non-bleached rhodopsin in intact retinas and living frogs. J. Biol. Chem..

[43] Gilliland CT, Salanga CL, Kawamura T, Trejo J, Handel TM (2013). The chemokine receptor CCR1 is constitutively active, which leads to G protein-independent, β-arrestin-mediated internalization. J. Biol. Chem..

[44] Kang Y, Zhou XE, Gao X, He Y, Liu W, Ishchenko A, Barty A, White TA, Yefanov O, Han GW, Xu Q, de Waal PW, Ke J, Tan MH, Zhang C, Moeller A, West GM, Pascal BD, Van Eps N, Caro LN, Vishnivetskiy SA, Lee RJ, Suino-Powell KM, Gu X, Pal K, Ma J, Zhi X, Boutet S, Williams GJ, Messerschmidt M, Gati C, Zatsepin NA, Wang D, James D, Basu S, Roy-Chowdhury S, Conrad CE, Coe J, Liu H, Lisova S, Kupitz C, Grotjohann I, Fromme R, Jiang Y, Tan M, Yang H, Li J, Wang M, Zheng Z, Li D, Howe N, Zhao Y, Standfuss J, Diederichs K, Dong Y, Potter CS, Carragher B, Caffrey M, Jiang H, Chapman HN, Spence JC, Fromme P, Weierstall U, Ernst OP, Katritch V, Gurevich VV, Griffin PR, Hubbell WL, Stevens RC, Cherezov V, Melcher K, Xu HE (2015). Crystal structure of rhodopsin bound to arrestin by femtosecond X-ray laser. Nature.

[45] Zhou XE, He Y, de Waal PW, Gao X, Kang Y, Van Eps N, Yin Y, Pal K, Goswami D, White TA, Barty A, Latorraca NR, Chapman HN, Hubbell WL, Dror RO, Stevens RC, Cherezov V, Gurevich VV, Griffin PR, Ernst OP, Melcher K, Xu HE (2017). Identification of phosphorylation codes for arrestin recruitment by G protein-coupled receptors. Cell.

[46] Gurevich VV, Gurevich EV (2018). GPCRs and signal transducers: Interaction stoichiometry. Trends Pharmacol. Sci..

[47] Smith JS, Pack TF, Inoue A, Lee C, Zheng K, Choi I, Eiger DS, Warman A, Xiong X, Ma Z, Viswanathan G, Levitan IM, Rochelle LK, Staus DP, Snyder JC, Kahsai AW, Caron MG, Rajagopal S (2021). Noncanonical scaffolding of Galphai and β-arrestin by G protein-coupled receptors. Science.

[48] Nagai T, Ibata K, Park ES, Kubota M, Mikoshiba K, Miyawaki A (2002). A variant of yellow fluorescent protein with fast and efficient maturation for cell-biological applications. Nat. Biotechnol..

