# Supplementary material for: Agonist dependency of the second phase access of β-arrestin 2 to the heteromeric µ-V1b receptor
Source: Sci Rep. 2021 Aug 4;11:15813. doi: 10.1038/s41598-021-94894-y (PMC8339129; doi:10.1038/s41598-021-94894-y)

Supplemental material

# Agonist dependency of the second phase access of β-arrestin 2 to the heteromeric μ-V1b receptor

Nuttawadee Ngamlertwong, Hiroyoshi Tsuchiya, Yuta Mochimaru, Morio Azuma, Takahiro Kuchimaru and Taka-aki Koshimizu[*]

[*]Correspondence; t_koshi@jichi.ac.jp
Division of Molecular Pharmacology, Department of Pharmacology, Jichi Medical University, Tochigi 329-0498, Japan

Figure Legends of Supplementary Figures

Figure S1. Positive NanoBRET between receptor homodimers in the plasma membrane, but not between unrelated receptor types. The μ-type opioid or V1b receptor, which was fused to Nluc or Venus and P2X2a purinergic channel was fused to Venus. Combinations of receptor and channel genes were expressed in HEK cells. Luminescence signal at 530/480 nm was measured for 5 min. The data was averaged and plotted with mean and S.E.M. values. (n=12)

Figure S2. The development of luminescence signals from whole luciferase formed by LgBit and SmBit. HEK cells expressing V1a-LgBit and V1a-SmBit (a) or membrane samples extracted from these cells (b) were stimulated with 100 nM AVP or control buffer for 5 min. After the stimulation period, the time course of the luminescence signal was recorded for 5 min at 480 nm after the addition of a luminescence substrate. In panel (c), cAMP-dependent protein kinase type II-alpha regulatory subunit and catalytic subunit pairs were used for constitutive dimer. (n=13, 9 and 10 for a, b, and c, respectively)

Figure S3. Co-expression of Nluc-connected receptor and native receptor at high expression levels did not show a co-operative increase in BRET signal. μ- or V1b-Nluc (0.75 μg/35 mm dish) was transfected with V1b or μ receptor (0.75 μg/35 mm dish) and β-arrestin 2-Venus (1.5 μg/35 mm dish). BRET measurements were performed under stimulation of DAMGO and AVP as indicated.

Figure S4. Detection of μ-Nluc by western blot analysis. HEK cells in 10 cm dishes at $10^6$ cells/dish were transfected with specified amounts of the plasmid for HA-tagged μ-Nluc. The plasmid amounts used in a 10 cm dish were about 8 times more than those used for transfection in a 35 mm dish (0.75–0.005 μg/35 mm dish). The cells were grown for 48 h and collected into lysis buffer placed on ice. Lysate was centrifuged and HA-tagged μ-Nluc in the supernatant was immunoprecipitated with anti-HA antibody. Western blot proteins were detected with HA-antibody and visualized by HRP-labeled secondary antibody and chemiluminescent procedures. The expected molecular weight of the HA-tagged μ-Nluc was 72.3 kDa, as indicated by the scale bar to the right side of the panel. Experiments were repeated twice and yielded similar results.

# Supplemental Figure 1

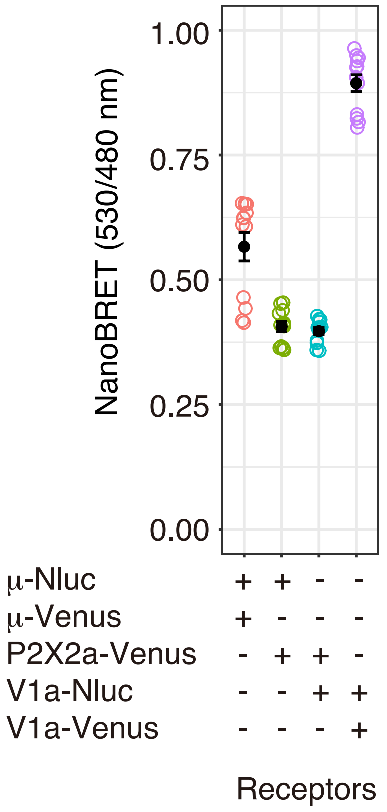

# Supplementary Figure 2

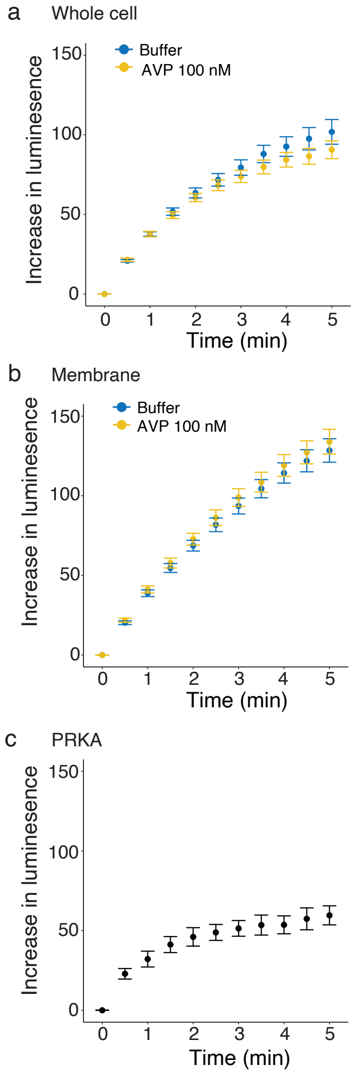

# Supplemental Figure 3

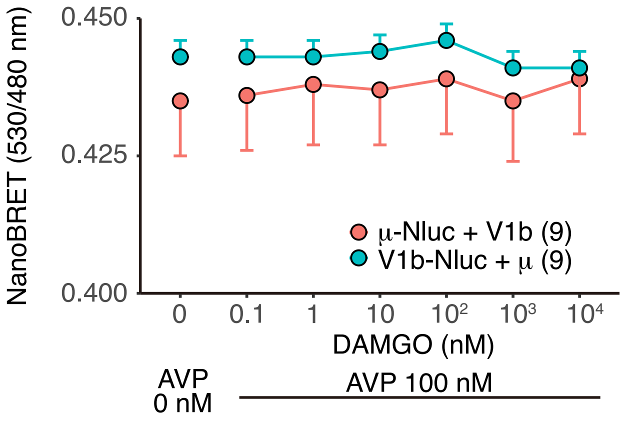

Supplemental Figure 4

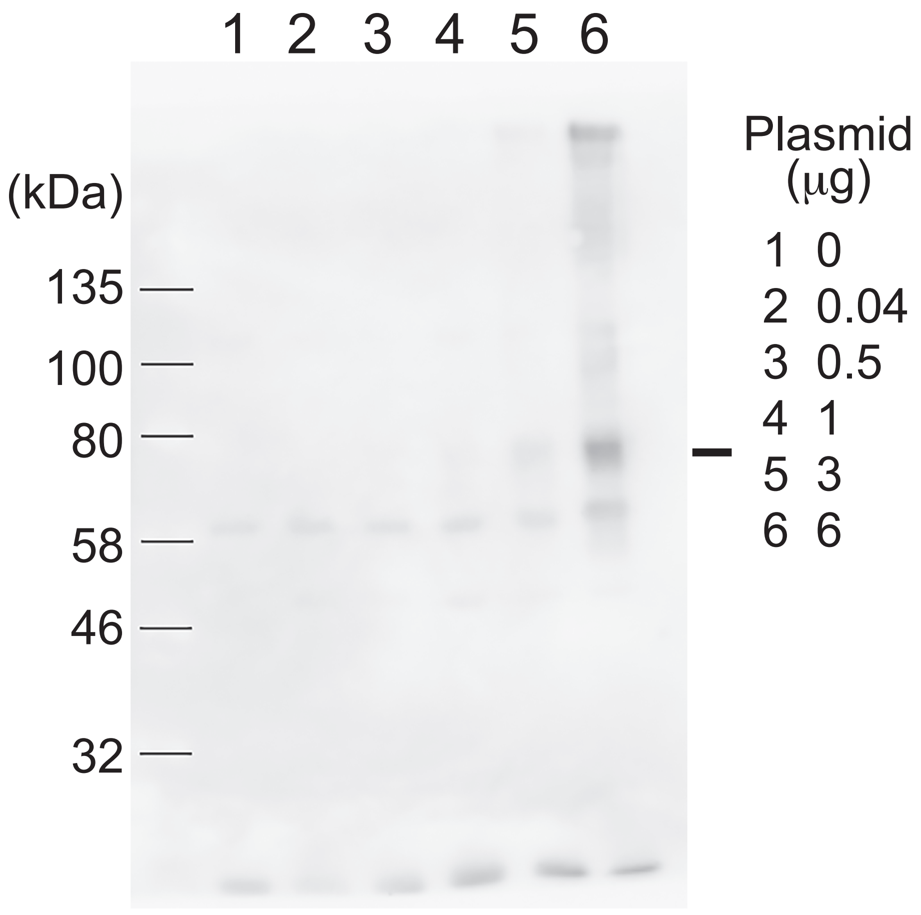

Supplement: Supplementary file 1 — Supplementary Information. [file 41598_2021_94894_MOESM1_ESM.pdf]
